# Pyrrolidine dithiocarbamate accelerates early recovery of intestinal microvascular oxygenation in a reversible model of hemorrhagic shock in rats

**DOI:** 10.1186/s12967-025-07413-2

**Published:** 2025-11-13

**Authors:** Stefan Hof, Leandra Krüll, Jeanne Schmitt, Christopher Neumann, Carsten Marcus, Anne Kuebart, Anna Herminghaus, Borna Relja, Christian Vollmer, Inge Bauer, Olaf Picker, Richard Truse

**Affiliations:** 1 Department of Anesthesiology, University Hospital Duesseldorf, Duesseldorf, Germany; 2 Department of Anesthesia, Critical Care and Pain Medicine, Beth Israel Deaconess Medical Center, Harvard Medical School, Boston, MA USA; 3 Department of Trauma, Hand, Plastic and Reconstructive Surgery, Translational and Experimental Trauma Research, University Hospital Ulm, Ulm, Germany

**Keywords:** PDTC, Heme oxygenase-1, HO-1, µHbO_2_, Hemorrhagic shock, Intestine

## Abstract

**Background:**

Improving intestinal tissue integrity appears to be crucial for maintaining global homeostasis in patients suffering from hemorrhagic shock. Since depletion of regional oxygen reserve might lead to cell death and tissue injury, the maintenance of regional tissue oxygenation appears to prevent intestinal mucosa from barrier shut down. Pyrrolidine dithiocarbamate (PDTC), an inducer of heme oxygenase-1 (HO-1), was reported to improve tissue integrity in various tissues under conditions of restricted oxygen delivery. Therefore, we investigated the effect of PDTC pretreatment on intestinal microvascular oxygenation (µHbO_2_) as a measure of regional tissue oxygen reserve in a rat model of hemorrhagic shock with subsequent shed blood transfusion. Furthermore, we explored whether the effect of systemic PDTC pretreatment on intestinal µHbO_2_ is associated with alterations in microvascular and mitochondrial function, as well as heme oxygenase-1 (HO-1) protein expression in the intestine.

**Methods:**

40 male Wistar rats were randomized into 4 experimental groups and received standardized instrumentation under general anesthesia. Hemorrhagic shock was induced by arterial blood withdrawal (MAP: 40 ± 5 mmHg; 1 h). Subsequently, shed blood was transfused and animals were observed for 2 h. Control animals were observed for 3 h without the induction of hemorrhagic shock. PDTC (100 mg·kg^− 1^, i.p.) or saline was applied 24 h prior to hemorrhagic shock or control treatment. µHbO_2_ of the ileum and the colon was continuously evaluated by white-light spectrophotometry. Variables of microvascular blood flow, microvascular diffusion distance and mitochondrial respiration were determined by laser Doppler flowmetry as well as incident dark-field imaging and respirometry. HO-1 protein expression was assessed by western blot analysis.

**Results:**

Hemorrhagic shock decreased intestinal µHbO_2_. Whereas systemic PDTC application did not increase µHbO_2_ under physiological conditions, pharmacological pretreatment significantly improved colonic µHbO_2_ after 60 min of hemorrhagic shock and accelerated early recovery of ileal and colonic µHbO_2_. This observation was independent of microvascular perfusion and mitochondrial respiration. PDTC pretreatment led to an increase of relative HO-1 protein expression in the ileum without a significant effect on colonic protein expression.

**Conclusion:**

The gastrointestinal tract is at persistent risk to develop tissue injury during hemorrhagic shock. The systemic application of PDTC is a promising pharmacological strategy to improve intestinal oxygen reserve during hemorrhagic shock and subsequent blood transfusion. This might lead to reduced intestinal tissue injury, maintain global homeostasis and reduce morbidity of patients suffering from hemorrhagic shock. However, the exact mechanism by which PDTC pretreatment improves intestinal oxygen reserve has to be elucidated in further studies since neither microvascular nor mitochondrial function was altered after PDTC application in this study. Furthermore, observed effects might include both HO-1-dependent and HO-1-independent mechanisms.

## Introduction

Hemorrhagic shock is a life-threatening event with a relevant impact on the patients outcome [1]. However, the pathophysiological mechanisms of hemorrhagic shock induced tissue injury are not completely understood and therapeutic approaches to preserve tissue integrity in patients with acute bleeding are lacking. In cases of fulminant hemorrhage, hemodynamic failure and, in traumatic scenarios, severe brain injury account for most deaths within the first few hours. In contrast, late morbidity is dominated by systemic inflammation, sepsis and multi organ failure [2]. These inflammatory states can only be partially attributed to exogenous infections and are more likely the result of disrupted physiological barriers and the inflammatory response to tissue damage [3]. In this context, the maintenance of intestinal integrity could be an additional goal for therapeutic approaches aiming to avoid secondary diseases for several reasons [4]. First, the gastrointestinal surface is one of the largest surfaces of the human body and exposed to a plurality of commensal bacteria [5]. In the case of intestinal barrier disruption, the translocation of enterobacteria and pathogen-associated molecules therefore might induce systemic inflammation [4]. Second, the gastrointestinal tract is crucial to establish a balance between pathogen defense and immune tolerance [6]. Disturbance of this immunological balance might induce a sterile and dysfunctional activation of immune cells. Third, gastrointestinal microcirculation is impaired immediately after the onset of hemorrhagic shock as a consequence of blood volume redistribution [7]. This leads to pronounced tissue damage of the intestine during hemorrhagic shock. Although the maintenance of intestinal tissue integrity appears to be pivotal in the prevention of secondary diseases after hemorrhage and trauma, concepts for intestinal tissue protection as a supportive therapeutic strategy alongside acute bleeding control and the administration of blood products are lacking.

Heme oxygenase-1 (HO-1) - also known as heat shock protein 32 - was reported to induce potent tissue protection during hemorrhagic shock in the lungs [8, 9] the kidneys [10] and the intestine [11] and therefore appears to be a promising target for pharmacological interventions to maintain tissue integrity during shock. Contrary to its isoform HO-2, HO-1 is inducible by adverse stimuli [12] and plays a key role in the resistance to oxidative stress and inflammation [13]. Moreover, HO-1 may also play a crucial role under physiological conditions, as early studies have shown increased intrauterine mortality in animals lacking functional HO-1 [14]. There are two relevant mechanisms by which HO-1 leads to its subordinated effects. First, HO-1 is the pacemaker enzyme of oxidative heme catabolism, which leads to the release of biliverdin, carbon monoxide and elemental iron [15]. Unbound heme is toxic and acts as a pro-oxidant molecule [16]. Removing heme therefore is likely to reduce cell stress. In addition, immediate effects of the released products of heme degradation contribute to tissue protection [17]. Second, HO-1 is translocated to the nucleus under adverse conditions to exert non-enzymatic effects such as amplifying its own expression or regulating transcriptional factors related to oxidative stress [18].

Hemorrhagic shock is characterized by a reduction of systemic oxygen supply, that does not meet the physiological requirements of basal energy metabolism of functional tissues [19]. Therefore, improving tissue oxygenation appears to be crucial for protecting organs from injury. Mitochondrial function and regional microcirculation are major determinants of tissue oxygenation and were defined as the critical unit in critically ill patients [20]. Targeting mitochondrial and microcirculatory variables for pharmacological resuscitation therefore could be suitable to improve tissue homeostasis in an early stage of regional derangement mitigating tissue damage after hemorrhagic shock and reperfusion. Several HO-1 related effects and especially those related to oxidative stress [21] and cell death [22] are linked to the mitochondria. Further, HO-1 can be translocated not only to the nucleus but also to the mitochondrial matrix leading to cell protection in renal epithelial cells [23] and the gastrointestinal tract [24]. This supports the assumption that induction of HO-1 improves mitochondrial respiration, as also described in the liver [25] and cultured lung epithelial cells [26]. Nitric oxide, a potent mediator of the microcirculation that was previously reported to improve microvascular oxygenation of gastric mucosa in a canine model of hemorrhagic shock [27, 28], appears to play a pivotal role in the posttranscriptional regulation of HO-1 signaling in vascular smooth muscle cells [29]. Likewise, nitric oxide stimulates HO-1 expression [30]. In turn, carbon monoxide generated by HO-1 promotes the production of cyclic guanosine monophosphate (cGMP), similar to nitric oxide, and activates potassium channels, resulting in precapillary vasodilation [31]. This intensive and synergistic cross-talk between nitric oxide and carbon monoxide was described to form a functional unit in health and disease [32] giving the possibility to improve regional microcirculation of the liver [33] and the intestine [34] during hemorrhagic shock. However, in a canine model of hemorrhagic shock neither direct intravenous application nor inhaled carbon monoxide exerted beneficial effects on gastric tissue oxygenation [35].

Synoptically, mitochondrial and microcirculatory effects of HO-1 induction during hemorrhagic shock are insufficiently described in the intestinal tract. However, pharmacological induction of HO-1 could improve microvascular and mitochondrial function as a supportive therapeutic strategy to optimize tissue oxygen supply and reduce multiple organ failure in critically ill patients. Therefore, this study was designed to investigate the impact of pyrrolidine dithiocarbamate (PDTC), a potent HO-1 inducer, on ileal and colonic tissue oxygenation, mitochondrial function and microcirculatory variables. Further, lipid peroxidation as a consequence of oxidative stress as well as functional and structural damage of the intestine after acute hemorrhage-reperfusion injury was determined as secondary endpoints.

## Materials and methods

All experiments were approved by the local Animal Care and Use Committee (Landesamt für Natur, Umwelt und Verbraucherschutz, Recklinghausen, Germany, AZ. 81-02.04.2018.A308) and performed in accordance with the ARRIVE guidelines for animal care. Animals were derived from the animal research facility of the Heinrich-Heine-University Duesseldorf (ZETT, Zentrale Einrichtung für Tierforschung und wissenschaftliche Tierschutzaufgaben) and bred for experimental purposes.

**Fig. 1:**
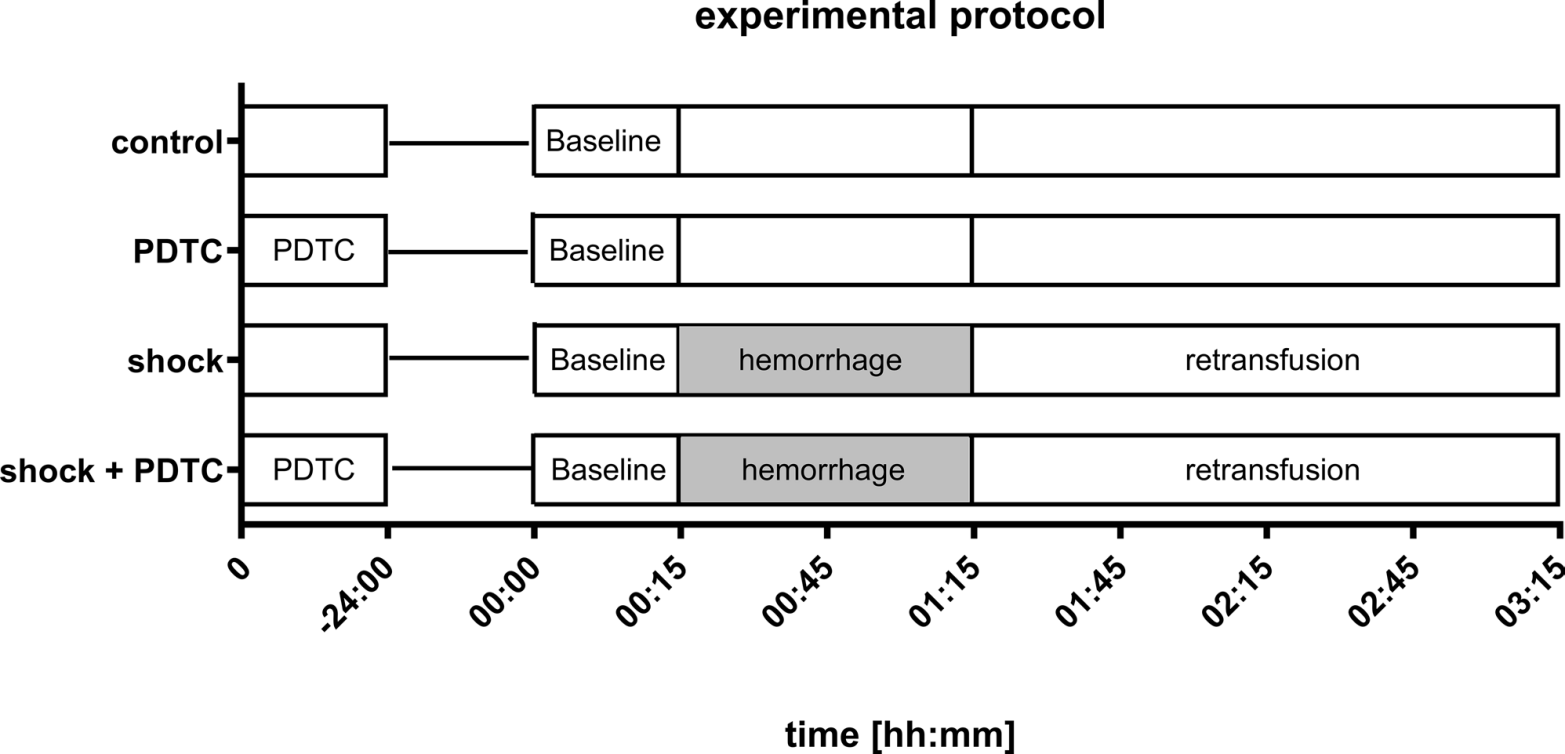
Experimental protocol. Animals were anesthetized and underwent standardized instrumentation. After registration of baseline values, hemorrhagic shock was induced by intermittent arterial blood withdrawal (MAP: 40 ± 5 mmHg; 1 h). Hemorrhagic shock was followed by shed blood transfusion and an observation period of 2 h. Control animals were anesthetized and catheterized equally and were observed for 3 h without the induction of hemorrhagic shock. 24 h before the induction of hemorrhagic shock, pyrrolidine dithiocarbamate (PDTC; 100 mg·kg^− 1^) was injected into the peritoneal cave under light sedation. Control animals received saline with an equivalent injection volume

40 male Wistar rats (350 ± 35 g body weight) were randomized into four groups with 10 individuals each (Fig. 1: Experimental protocol).

**Fig. 2:**
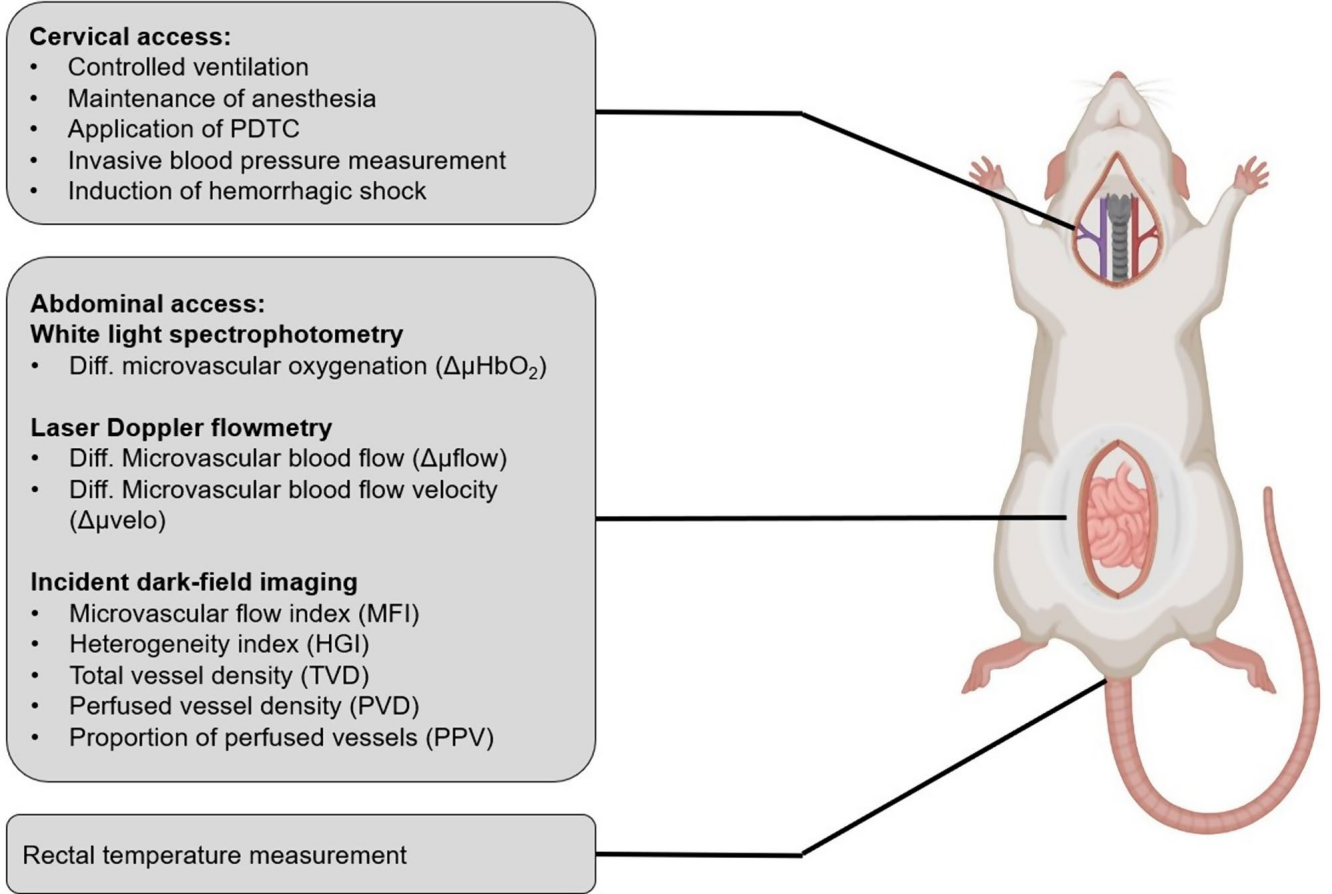
Experimental instrumentation. Schematic overview of the surgical instrumentation and the microcirculatory measurements used in the in vivo experiments. Microcirculatory variables of the intestine are assigned to the corresponding measuring principle. Some components of this figure were created with BioRender

Under conditions of light sedation (sodium pentobarbital 40 mg·kg^− 1^ body weight i.p.) pyrrolidine dithiocarbamate (PDTC; 100 mg·kg^− 1^ body weight i.p.) was injected into the peritoneal cave. Saline was applied in control animals that did not receive PDTC treatment. The investigator was blinded to the substance applied. After 24 h animals were anesthetized again (sodium pentobarbital 100 mg·kg^− 1^ i.p. followed by 10 mg·kg^− 1^·h^− 1^ i.v.) and received standardized instrumentation via a cervical surgical access consisting of tracheostomy, an invasive arterial blood pressure measurement and a central venous catheterization. After median laparotomy the ileocecal junction region was gently exposed for continuous and intermittent microcirculatory measurement in vivo (Fig. 2: Experimental instrumentation).

Carbon dioxide [36] and mild hypothermia [37] were reported to modulate microvascular variables during hemorrhagic shock in dogs. Therefore, intermittent blood gas analysis was used to ensure normocapnic ventilation. Likewise, animals were placed on a heating plate and body temperature was determined using a rectal probe to maintain normothermia. After the registration of steady-state conditions, that were defined as the stability of macro- and microcirculatory variables for at least 10 min, baseline values were recorded. Then, a hemorrhagic shock was established by removing blood via the arterial catheter. Blood was taken until a mean arterial blood pressure (MAP) of 40 ± 5 mmHg was reached. This level of blood pressure depression was maintained by intermittent blood withdrawal for one hour. Meanwhile, the shed blood was stored with citrate-phosphate-dextrose (Santa Cruz biotechnology Inc., Dallas USA). Then, the taken blood was filtered to retract residual blood clots and retransfused to the animals (Sangofix, pore size: 200 μm, B. Braun Melsungen, Germany). Animals that served as normovolemic controls did not undergo hemorrhage and shed blood transfusion, but were observed under general anesthesia for equal time periods. After the experiments all animals were euthanized by exsanguination in deep anesthesia (additional injection of 15 mg · kg^− 1^ i.v. sodium pentobarbital). Post mortem, ileal and colonic tissue samples were collected to determine mitochondrial respiration ex vivo. Likewise, tissue samples and blood plasma were collected and stored at -80 °C for further analysis.

### HO-1 protein expression

HO-1 protein expression in colon and ileum of animals with and without PDTC pretreatment was determined in a subset of the experiments by Western blot analysis. Tissue was homogenized in RIPA Lysis Buffer (150 mM NaCl, 1.0% NP-40, 0.5% deoxycholate, 0.1% SDS, 50 mM Tris-Cl). Samples were centrifuged for 5 min at 16,000 g and 4 °C. Protein concentration was determined in the supernatant with PierceTM BCA protein assay kit (Thermo Scientific, Rockford, IL, USA). Aliquots of proteins (25 µg of protein per lane) were separated under denaturing conditions by SDS PAGE (12%) and transferred (Power Blotter XL; Invitrogen by Thermo Fisher Scientific; Carlsbad, CA, USA) to polyvinylidene difluoride (PVDF) membranes (0.45 μm, Immobilon-P Transfer Membrane; Millipore Merck, Darmstadt, Germany). After blocking unspecific binding sites with 5% bovine serum albumin in Tris-buffered saline/Tween (TBS-T; 20 mmol/L Tris [pH 7.5], 0.5 mol/L NaCl, 0.1% Tween 20), membranes were incubated with anti-HO-1 antibody (HO-1 rabbit monoclonal antibody, 32 kDa, Abcam #189491; dilution 1:1000; Abcam, Cambridge, UK) followed by a secondary antibody (IRDye 680LT, LICORbio #926-68020, dilution 1:15000; LICORbio Bad Homburg, Germany) for photometric detection. HO-1 signal was set in relation to β-actin, that was visualized with an anti-β-actin antibody (β-actin mouse monoclonal antibody, 45 kDa, Cell Signaling. #3700; dilution 1:15000; Cell Signaling Technologies, Danvers, MA, USA) and subsequent incubation with the secondary antibody IRDye 800CW (LICORbio #926-32211; dilution 1:15000; LICORbio Bad Homburg, Germany). Hepatic tissue samples served as positive controls [38]. Signals were detected with LI-COR odyssey XF 2800 imaging system (LI-COR Biosciences; Bad Homburg, Germany) and Image Studio Software 5.1. Quantification was performed with Software Empiria Studio version 2.3 (LI-COR Biosciences; Bad Homburg, Germany).

### Microvascular oxygenation and microcirculation

#### Reflectance spectrophotometry and laser doppler flowmetry

White light consisting of homogeneously distributed wavelengths between 450 and 1000 nm was generated by a separated light source to illuminate intestinal tissue in-vivo. According to the spectral properties of hemoglobin, the emitted light is wavelength-dependently absorbed by erythrocytes or scattered at cellular structures and interstitial matrix components. Therefore, the reflected signal differs from the emitted white light in terms of intensity and wavelength spectrum. In the here used device (O2C LW 2222, LEA Medizintechnik GmbH, Gießen, Germany) one probe (Flat Probe LFX-2, LEA Medizintechnik GmbH, Gießen, Germany) was used for both, light emission and light detection. Thereby, the change of light intensity is inversely proportional to the relative amount of hemoglobin per unit tissue volume. The averaged microvascular oxygenation (µHbO_2_; [%]) was determined by analyzing the reflected wavelength spectrum and comparing it to standard spectra of hemoglobin with distinct oxygen saturation grades. Since blood is predominantly stored in the venous compartment of microcirculation, the signal detected at the tissue surface is shifted and mainly indicates postcapillary oxygen saturation as a measure of regional oxygen reserve. In addition, a diode generated laser with 820 nm wavelength, integrated in the same device, was used to determine microvascular blood flow (µflow; [aU]) and blood flow velocity (µvelo; [aU]). When laser comes into contact with moving particles such as erythrocytes, the resulting frequency shift of the reflected signal can be used to calculate the averaged blood flow velocity according to the Doppler shift principle. Then, microvascular blood flow can be determined by further calculations [39]. Since microvascular vessels assembly as a three-dimensional network and the calculation of the Doppler shift depends on the angle between the emitted laser light and the vascular axis, both variables of microvascular perfusion are given in arbitrary units (aU) and are indexed as changes from baseline measurements (Δµflow; [aU] and Δµvelo; [aU]). Likewise, microvascular oxygenation in a defined section of the gastrointestinal tract might depend on the individual microvascular structure in this region with local and individual differences of microvascular oxygen reserve. Therefore, values of microvascular oxygenation are calculated as difference from baseline (ΔµHbO_2_ [%]).

White light spectrophotometry and laser Doppler flowmetry were performed by one device and common measuring probes, so that the obtained values refer to the identical tissue section. A simultaneous measurement via a second channel also allowed the measurement of other organ sections at the same time point. In this trial, the terminal ileum and the ascending colon were defined as regions of interest. Since vessels with a diameter larger than 100 μm result in total light absorption, only microvasculature is detected by this method [40].

#### Videomicroscopy

The microcirculation of the ascending colon was intermittently visualized by incident dark field (IDF) imaging (CytoCam, Braedius Medical, Huizen, The Netherlands). For this purpose, the examined tissue was illuminated with pulsed green light of 530 nm wavelength corresponding to the isosbestic point of oxygenated and deoxygenated hemoglobin. Hemoglobin, regardless of the degree of oxygenation, results in complete extinction of green light, while interstitial cells and matrix components result in light reflection. Thus, erythrocytes become visible as black light recesses. Using a complex lens system, different depths of the investigated tissue can be visualized optically. The resulting video sequences were stabilized, checked for sufficient quality, and further processed, blinded and randomized. Only videos that met the requirements of an international consensus panel were included in the study [41, 42]. Using an automated software (MicroCirculation Analysis software, Braedius Medical, Huizen, The Netherlands), the total vessel density (TVD), the perfused vessel density (PVD) and the percentage of perfused vessels (PPV) were calculated. In addition, the microvascular flow index (MFI) was determined by a blinded investigator as a measure of microvascular blood flow quality according to its first description by Spronk et al. [43]. This score has been validated by Boerma et al. for different parts of the gastrointestinal tract [44]. Heterogeneity index (HGI) was determined a measure of spatial blood flow distribution in a plane microvascular layer [45]. All videos were determined in close proximity to the microcirculation measurement by white light spectrophotometry and laser Doppler flow measurement.

### Mitochondrial respiration and oxidative stress

#### Respirometry

Tissue samples of ileum and colon were harvested at the end of the experiments and homogenates were prepared like described previously [46]. Then, respirometry by using a Clark-type electrode (model 782, Strathkelvin instruments, Glasgow, Scotland) was performed to measure mitochondrial respiration. Complex I and complex II of the respiratory chain were stimulated separately by the supplementation with either glutamate (Fluka Chemie GmbH Buchs, Switzerland, 2.5 mM) and malate (Serva Electrophoresis GmbH, Heidelberg, Germany, 2.5 mM) or succinate (Sigma-Aldrich Corporation, St. Louis, MO, USA, 5 mM), respectively. To target respiratory function exclusively via complex II, complex I activity was inhibited by rotenone application (0.5 µM, Sigma-Aldrich Corporation, St. Louis, USA). 2.5 µM cytochrome c and 0.05 µg·ml^− 1^ oligomycin were used to exclude mitochondrial leaking of the outer and inner membrane. State 2 of oxidative phosphorylation – residual oxygen consumption for compensation of proton leak – was measured after addition of substrats for complex I or II. If adenosine diphosphate (Sigma-Aldrich Corporation, St. Louis, MO, USA, 125 µM) is added, mitochondrial respiration increases to maximal respiratory capacity (state 3) until ADP is completely metabolized (state 4). Respiration rates are expressed as nanomole oxygen consumption per minute per milligram protein (nmol·min^− 1^·mg^− 1^). Finally, respiratory control index (RCI = state 3 / state 2), indicating mitochondrial coupling between the respiratory chain and the oxidative phosphorylation, and ADP/O-ratio (ADP/O-ratio = ADP added / O_2_ consumption in state 3) as an index of respiratory efficiency were calculated.

#### Malondialdehyde levels

If polysaturated fatty acids are degraded by lipid peroxidation malondialdehyde (MDA) is generated and indicates oxidative stress. Tissue samples of ileum and colon were harvested at the end of the experiments to determine intestinal MDA levels. First, frozen tissue was homogenized in 1.5% KCl (Fluka Chemie GmbH, Buchs, Switzerland) and mixed with 1% phosphoric acid (Merck, Darmstadt, Germany) and 0.6% thiobarbituric acid (Merck, Darmstadt, Germany). Then, the mixture was heated to 95 °C for 45 min and mixed with butanol (Merck, Darmstadt, Germany). MDA and thiobarbituric acid form a metabolite which is detected in the fluid supernatant spectrophotometrically at 535 nm and 520 nm. MDA concentration was normalized to tissue protein concentration. Values are reported as nanomole MDA per milligram protein.

### Intestinal tissue injury

#### Histological examination

Ileal tissue blocks were harvested, fixed with formaldehyde and embedded in paraffin blocks after the experiment for standard HE-staining (8 μm slides). Every slide was divided into five sections, which were scored separately by two blinded investigators using the Chiu-scoring system to access intestinal damage [47]. This scoring system focusses on gradual loss of adherence between enterocytes and the subepithelial stroma. All values were averaged to calculate the mean scoring value. Since the Chiu-scoring system is only validated for ileum slides, histological evaluation was not performed in tissue samples from the ascending colon. To assess spatial heterogeneity of intestinal tissue injury, histological heterogeneity index was calculated by dividing the difference between the maximum and minimum observed value by the mean value.

#### Plasmatic D-lactate concentration

In contrast to L-lactate, D-lactate is not produced in a relevant amount by mammalian cells but by endoluminal intestinal bacteria. Therefore, increasing levels of plasmatic D-lactate concentration might be a result of intestinal barrier dysfunction with increased mucosal permeability. A commercially available colorimetric assay (D-lactate, colorimetric assay kit, MAK058-1KT, Sigma-Aldrich, Germany) was used to determine plasmatic D-lactate concentration after hemorrhage and reperfusion. In detail, blood samples were taken at the end of the experiments and centrifuged (10 min, 7000 x g) to obtain blood plasma. Then, a colorimetric assay was used to determine MTT-formazan concentration in blood plasma at 450 nm wavelength.

#### Statistics

An a priori power analysis (G*Power Version 3.1.9.2 [48]) revealed a power of >0.8 for detection of differences between the different groups with *n* = 10 in 4 groups, α < 0.05 and η^2^ of 0.47 (calculated from previous experiments).

Data for micro- and macrocirculatory analysis were obtained during the preceding 5 min of each experimental period under steady-state conditions. Q-Q-plots were used to state normal distribution of the data (IBM SPSS Statistics 29, International Business Machine Corp., United States). Time-related differences and differences between the experimental groups were assessed by two-way analysis of variance for repeated measurements (ANOVA) combined with a Bonferroni post-hoc test (GraphPad Prism version 6.05 for Windows, GraphPad Software, La Jolla, CA, United States). D-lactate measurements, that were assessed once after the experiments and followed normal distribution, were analyzed using one-way analysis of variance and Šidák correction for multiple comparison. All data are presented as absolute values of mean ± standard error of the mean. Variables of mitochondrial respiration, MDA levels and histological scoring did not follow normal distribution and were analyzed using Kruskal-Wallis testing and Dunn´s multiple comparisons. Values are reported as median ± interquartile range for *n* = 10. Relative HO-1 expression was determined in a subset of the experiments with *n* = 6 and were analyzed using an unpaired two-tailed t-test between experimental groups.

## Results

### Mean arterial blood pressure

**Fig. 3:**
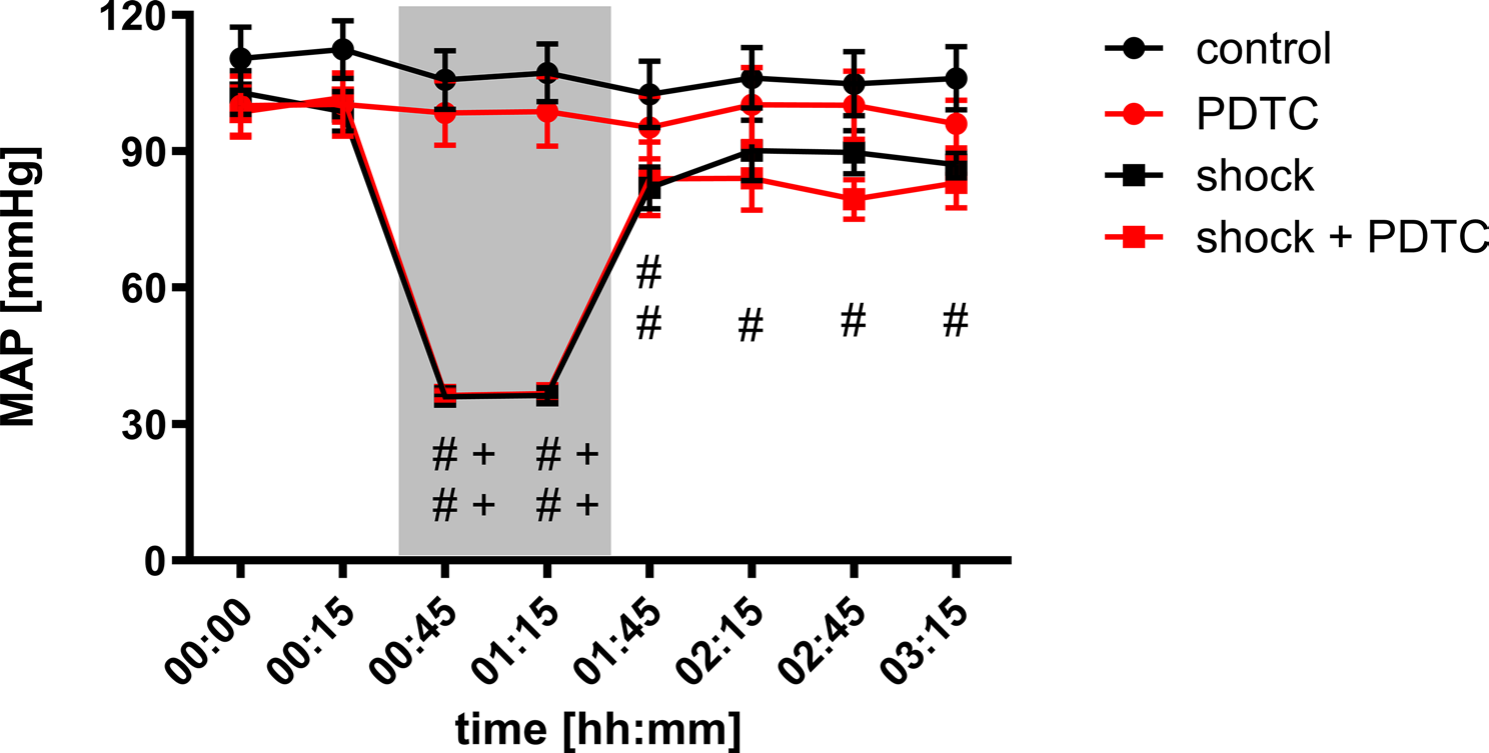
Mean arterial pressure. Changes of the mean arterial blood pressure (MAP; mmHg) over time under physiological and hemorrhagic conditions. Interventional groups received pyrrolidine dithiocarbamate (PDTC) pretreatment and/or fixed-pressure hemorrhage (shock). The period of acute hemorrhagic shock is marked grey. Data are presented as mean *±* SEM for *n* = 10 Wistar-rats. Statistics: 2-way ANOVA for repeated measurements followed by Bonferroni *post-hoc* test. # *p* ≤ 0.05 vs. individual baseline values, + *p* ≤ 0.05 vs. corresponding normovolemic control group

MAP values did not differ between experimental groups under baseline conditions and remained stable over time in normovolemic groups. PDTC pretreatment did not affect MAP under physiological conditions (control _00:00_: 110 ± 7 mmHg); PDTC _00:00_: 100 ± 7 mmHg). The induction of hemorrhagic shock led to a significant decrease of MAP compared to the individual baseline (shock _00:00_: 103 ± 5 mmHg; shock _00:45_: 36 ± 2 mmHg) and to the normovolemic control group (control _00:45_: 106 ± 6 mmHg). According to the experimental protocol of fixed-pressure hemorrhage, shock depth was independent of PDTC pretreatment (shock + PDTC _00:45_: 36 ± 1 mmHg). Transfusion of the shed blood reestablished baseline MAP levels in the shock group after 1 h (shock _02:15_: 90 ± 7 mmHg). In contrast, animals with PDTC pretreatment did not reach baseline MAP levels two hours after transfusion of the shed blood (shock + PDTC _03:15_: 83 ± 6 mmHg). However, there was no significant difference of MAP between the experimental groups in the intergroup analysis (Fig. 3: Mean arterial pressure) before and after the shock phase.

### HO-1 protein expression

**Fig. 4:**
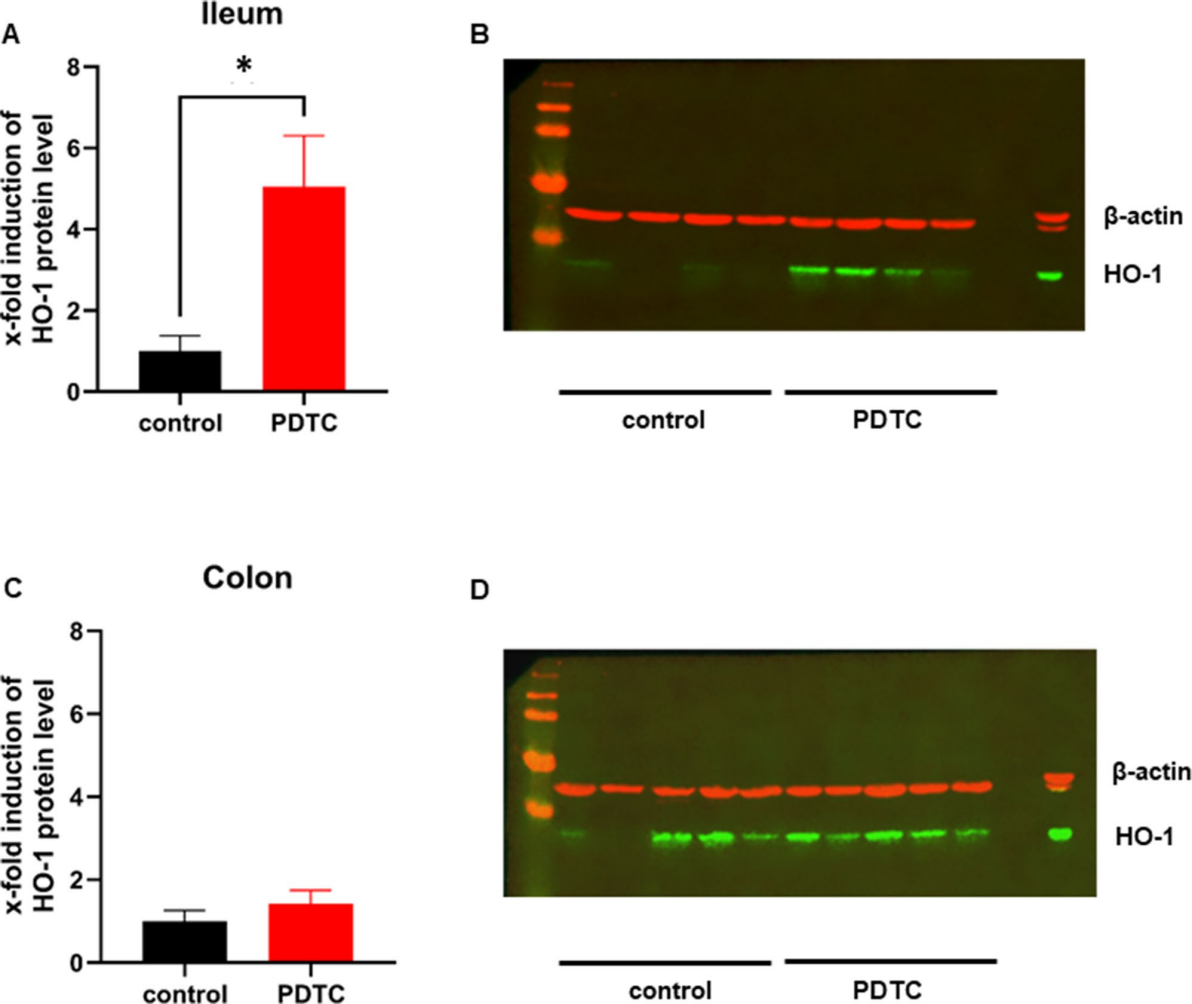
Heme oxygenase-1 protein expression. Ileal (**A**; **B**) and colonic (**C**; **D**) HO-1 protein expression was analyzed by Western blot analysis in animals that received either pyrrolidine dithiocarbamate (PDTC) or a control treatment. Protein expression was set in relation to β-actin and is indicated as relative protein induction compared to samples of animals without PDTC pretreatment (**A**; **C**), exemplary Western blots including positive control (right lane) are presented for ileum (**B**) and colon (**D**). Data are presented as mean ± SEM for *n* = 9 Wistar-rats. Statistics: Unpaired t-test, * *p* ≤ 0.05 vs. control group

The application of PDTC induces a 5-fold increase of ileal HO-1 protein expression (Fig. 4A and B). In the colon, PDTC application did not lead to a significant increase in HO-1 protein expression (Fig. 4C and D). Exemplary Western blots reveal a pronounced basal expression of HO-1 in the colon, but not in the ileum (B; D).

### Microvascular oxygenation

**Fig. 5:**
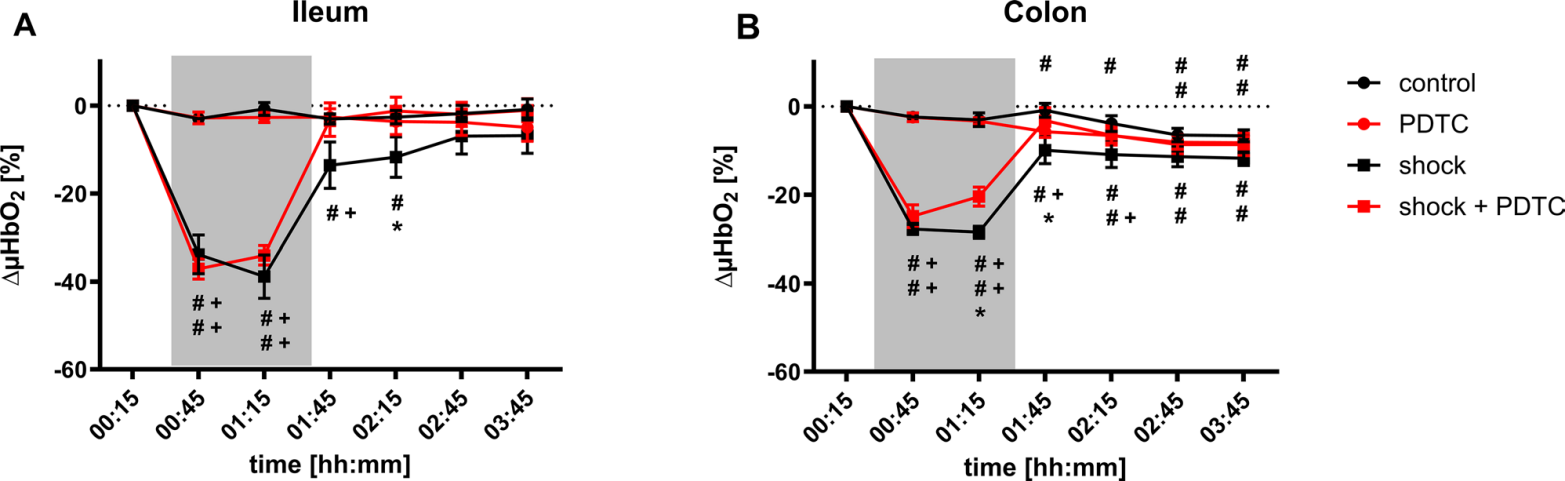
Microvascular oxygenation. Changes of ileal (**A**) and colonic (**B**) microvascular oxygenation (ΔµHbO_2_) in percentage [%] over time under physiological and hemorrhagic conditions. Interventional groups received pyrrolidine dithiocarbamate (PDTC) pretreatment and/or fixed-pressure hemorrhage (shock). The period of acute hemorrhagic shock is marked grey. Data are presented as mean *±* SEM for *n* = 10 Wistar-rats. Statistics: 2-way ANOVA for repeated measurements followed by Bonferroni *post-hoc* test. * *p* ≤ 0.05 vs. control group with the same hemodynamic conditions, # *p* ≤ 0.05 vs. individual baseline values, + *p* ≤ 0.05 vs. corresponding normovolemic control group

Whereas µHbO_2_ of the ileum remained stable over time in normovolemic animals, µHbO_2_ significantly decreased after the induction of hemorrhagic shock (shock _ileum; 00:45_: -34 ± 4%). In the colon of normovolemic animals, there was a slight but continuous decrease of µHbO_2_ over time that led to significantly lower µHbO_2_ values in the last hour of the experiments compared to baseline values (control _colon; 03:15_: -7 ± 1%). Further, the induction of hemorrhagic shock led to a pronounced decrease of colonic µHbO_2_ (shock _colon; 00:45_: -28 ± 1%). In colon and ileum changes during hemorrhagic shock were not only significant to the individual baseline, but also in relation to the normovolemic control group (control _ileum; 00:45_: -3 ± 1%; control _colon; 00:45_: -2 ± 1%). Prior application of PDTC had no effect on intestinal µHbO_2_ under physiological conditions, but ameliorated the decrease of colonic µHbO_2_ after 60 min of hemorrhagic shock (shock _colon; 01:15_: -29 ± 1% vs. shock + PDTC _colon; 01:15_: -21 ± 2%). Likewise, PDTC pretreatment accelerated the restoration of colonic µHbO_2_ immediately after transfusion of the shed blood (shock _colon; 01:45_: -10 ± 3% vs. shock + PDTC _colon; 01:45_: -3 ± 3%). In the terminal ileum µHbO_2_ values of the shock group without PDTC pretreatment did not recover completely within the first hour after transfusion (shock _ileum; 01:45_: -14 ± 5%; shock _ileum; 02:15_: -12 ± 5%). In contrast, baseline µHbO_2_ values of animals with prior PDTC treatment reestablished immediately (shock + PDTC _ileum; 01:45_: -3 ± 4%; shock + PDTC _ileum; 02:15_: -1 ± 3%) and were higher one hour after transfusion than values of animals without PDTC treatment. One hour after shed blood transfusion, PDTC pretreatment significantly improved µHbO_2_ values of the ileum in animals with prior shock induction (shock _ileum; 02:15_: -12 ± 5% vs. shock + PDTC _ileum; 02:15_: -1 ± 3%). Two hours after shed blood transfusion intestinal µHbO_2_ did not differ between experimental groups (Fig. 5: Differential microvascular oxygen saturation).

### Mitochondrial function

Intestinal respiration rates after selective complex I and complex II stimulation are reported in Table 1.

**Table 1:** Respiration rates. Electron transfer (state 2) and maximal mitochondrial respiration (state 3) were measured by respirometry in ileal and colonic tissue homogenates. Interventional groups received pyrrolidine dithiocarbamate (PDTC) pretreatment and/or fixed-pressure hemorrhage (shock). Data are presented as median (interquartile range) for *n* = 10 Wistar-rats. Statistical significance was accepted for *p* ≤ 0.05. Kruskal-Wallis testing and Dunn´s multiple comparisons did not reveal significant differences between experimental groups with or without PDTC treatment under the same hemodynamic conditions

	Groups	State 2	State 3
ileum complex I [nmol O_2_·min^− 1^·mg^− 1^]	control	0.42 (0.19)	0.72 (0.32)
	PDTC	0.42 (0.12)	0.73 (0.31)
	shock	0.35 (0.33)	0.64 (0.24)
	shock + PDTC	0.36 (0.30)	0.68 (0.26)
ileum complex II [nmol O_2_·min^− 1^·mg^− 1^]	control	0.44 (0.29)	0.92 (0.47)
	PDTC	0.44 (0.30)	0.85 (0.45)
	shock	0.39 (0.27)	0.79 (0.41)
	shock + PDTC	0.46 (0.27)	0.70 (0.20)
colon complex I [nmol O_2_·min^− 1^·mg^− 1^]	control	0.50 (0.30)	1.18 (0.78)
	PDTC	0.51 (0.21)	1.08 (0.70)
	shock	0.44 (0.34)	1.07 (0.50)
	shock + PDTC	0.49 (0.28)	1.00 (0.29)
colon complex II [nmol O_2_·min^− 1^·mg^− 1^]	control	0.63 (0.31)	1.78 (1.04)
	PDTC	0.68 (0.34)	2.01 (1.54)
	shock	0.45 (0.38)	1.68 (0.65)
	shock + PDTC	0.57 (0.17)	1.25 (0.50)

**Fig. 6:**
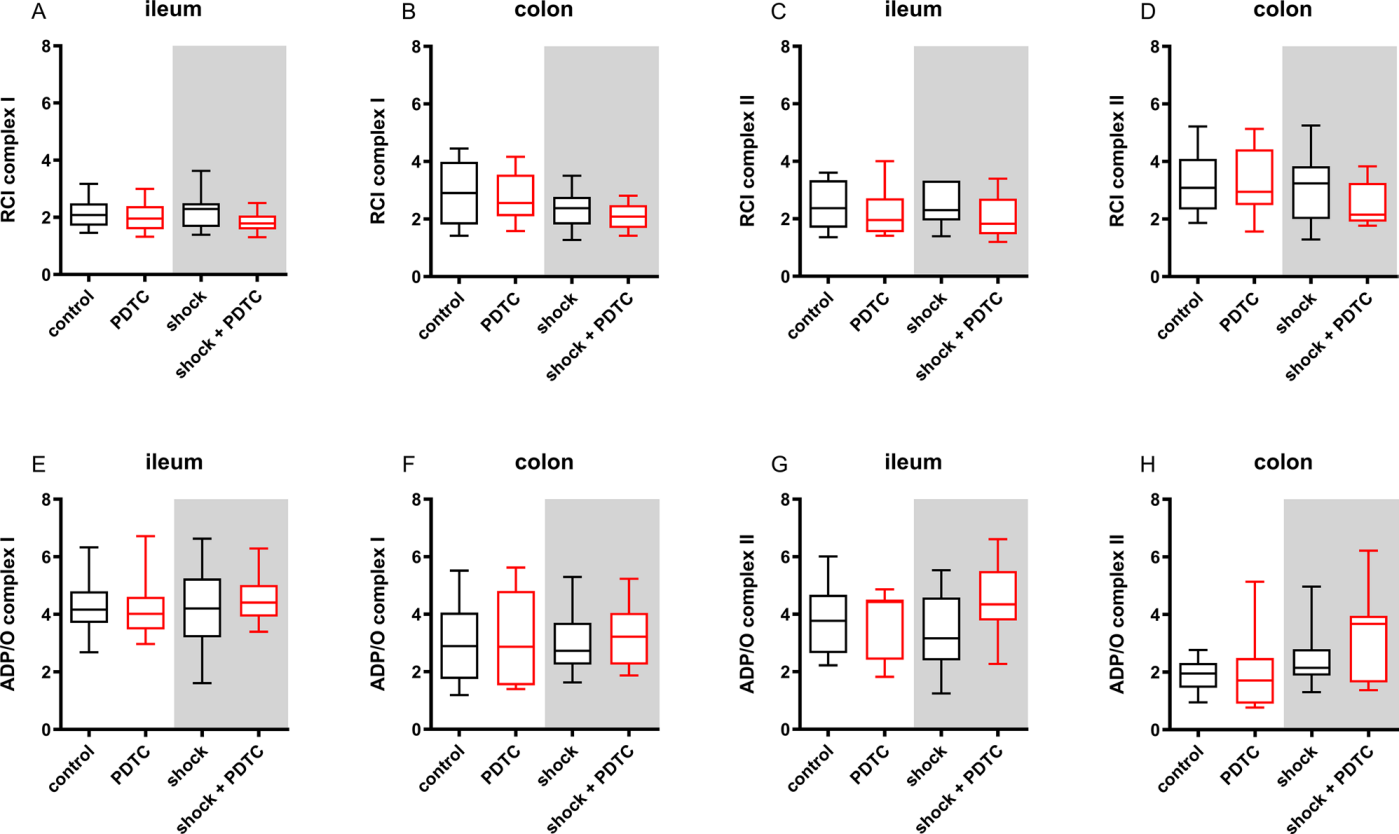
Integrative indices of mitochondrial function. Respiratory control index (RCI; **A**-**D**) and ADP/O-ratio (**E**-**H**) were used to determine mitochondrial coupling and respiratory efficiency in ileal and colonic tissue homogenates after stimulation of complex I (glutamate and malate) and complex II (succinate). Injection of pyrrolidine dithiocarbamate (PDTC; red boxplots) and/or a fixed-pressure shock (grey backing) was conducted *in*-*vivo* before the measurements were performed ex-vivo. Control animals received general anesthesia and standardized instrumentation without PDTC pretreatment and/or shock. Data are presented for *n* = 10. Whiskers indicate maximal and minimal values. Kruskal-Wallis testing and Dunn´s multiple comparisons did not reveal significant differences between the experimental groups

RCI and ADP/O-ratio served as integrative indices of mitochondrial function (Fig. 6: Integrative indices of mitochondrial function). RCI was 2.1 ± 0.8 in ileum samples of control animals and 2.9 ± 2.18 in colon samples after complex I stimulation. If complex II was stimulated, RCI was 2.4 ± 1.7 in the ileum and 3.1 ± 1.8 in the colon. Neither shock induction nor PDTC-treatment had an effect on RCI as a marker of mitochondrial coupling of the respiratory chain and the oxidative phosphorylation. Concordant to these findings on intestinal RCI, ADP/O-ratio was 4.2 ± 1.1 in ileum samples and 2.9 ± 2.3 in colon samples, if complex I of the control group was stimulated. Alternative stimulation via complex II led to ADP/O-ratios of 3.8 ± 2 in the ileum and 2 ± 0.9. in the colon. Neither acute hemorrhage nor PDTC treatment affected ADP/O-ratio after complex I or complex II stimulation.

### Oxidative stress

**Fig. 7:**
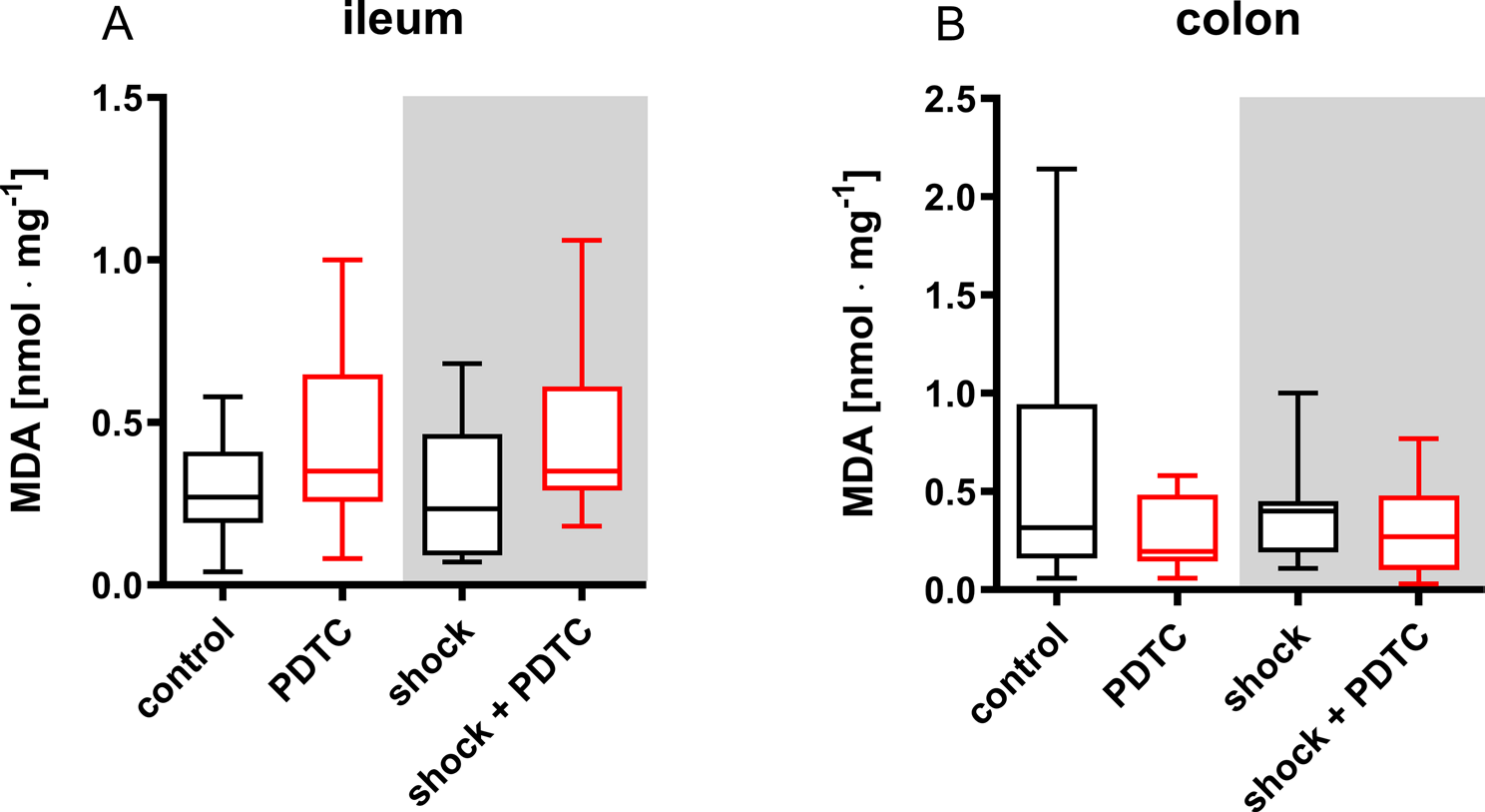
Tissue malondialdehyde concentration. Malondialdehyde concentration (MDA) of the ileum (**A**) and colon (**B**) was used as a marker of cellular lipid peroxidation. Pyrrolidine dithiocarbamate (PDTC) and/or a fixed-pressure shock was induced in vivo before the measurements were performed ex-vivo. Control animals received general anesthesia and standardized instrumentation without PDTC pretreatment and/or shock. Data are presented for *n* = 10 as median (interquartile range). Whiskers indicate maximal and minimal values. Kruskal-Wallis testing and Dunn´s multiple comparisons did not reveal significant differences between the experimental groups

Tissue MDA concentration of the control group was 0.27 ± 0.22 nmol · mg protein^− 1^ in the terminal ileum and 0.32 ± 0.79 nmol · mg protein^− 1^ in the ascending colon. In both sections of the gastrointestinal tract neither prior PDTC application nor the induction of hemorrhagic shock influenced tissue MDA concentration (Fig. 7).

### Microvascular perfusion

**Fig. 8:**
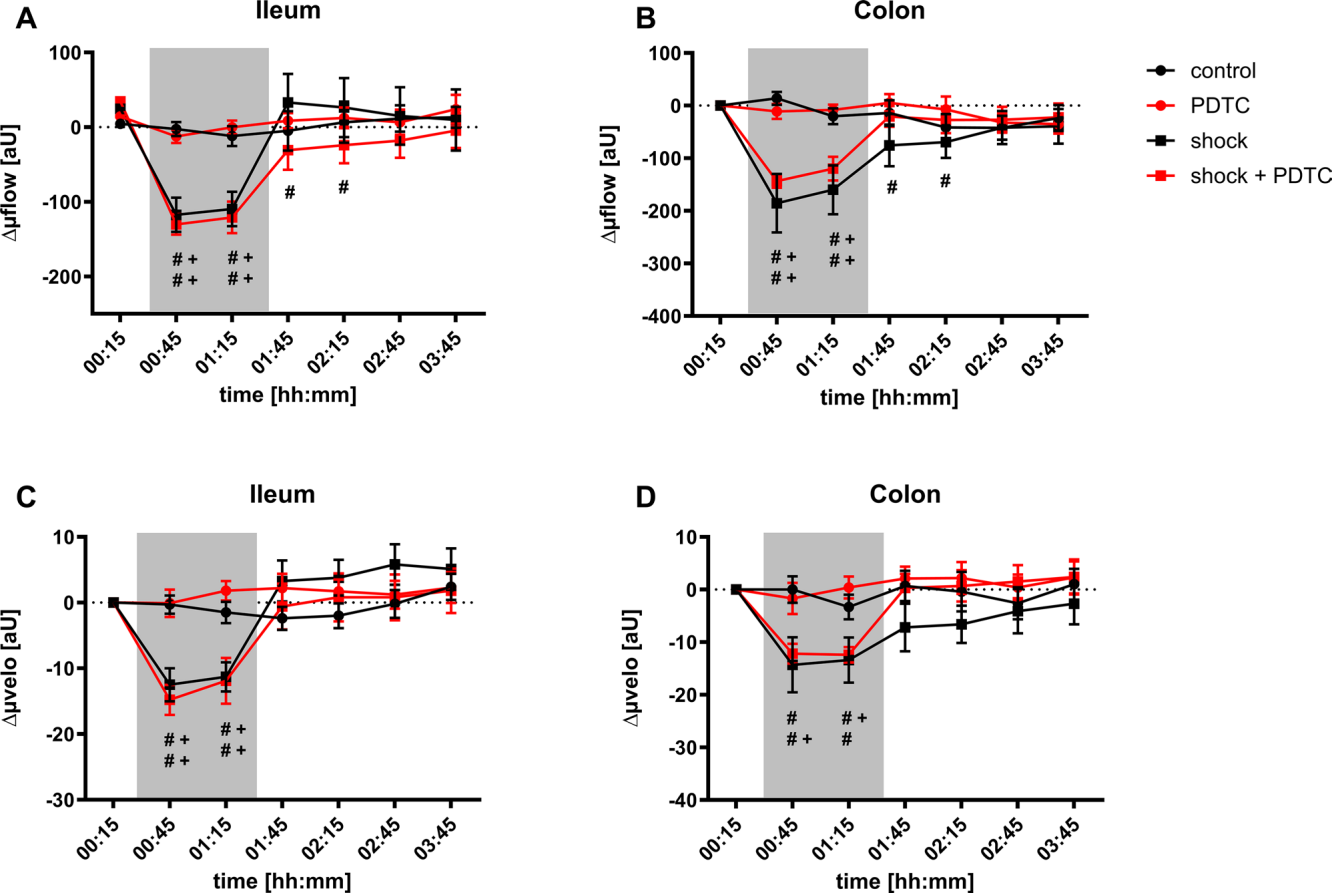
Microvascular perfusion. Changes of ileal (**A**) and colonic (**B**) microvascular blood flow (Δµflow) and ileal (**C**) and colonic (**D**) differential microvascular blood flow velocity (Δµvelo) in arbitrary units [aU] over time under physiological and hemorrhagic conditions. Interventional groups received pyrrolidine dithiocarbamate (PDTC) pretreatment and/or fixed-pressure hemorrhage (shock). The period of acute hemorrhagic shock is marked grey. Data are presented as mean *±* SEM for *n* = 10 Wistar-rats. Statistics: 2-way ANOVA for repeated measurements followed by Bonferroni *post-hoc* test. # *p* ≤ 0.05 vs. individual baseline values, + *p* ≤ 0.05 vs. the corresponding normovolemic control group

Intestinal µflow of normovolemic animals remained stable over time. Induction of hemorrhagic shock decreased µflow in the ileum (shock _ileum; 00:45_: -107 ± 23 aU) and in the colon (shock _colon; 00:45_: -186 ± 56 aU) compared to baseline values. These changes were also statistically significant compared to the normovolemic control group (control _ileum; 00:45_: -3 ± 9 aU; control _colon; 00:45_: 14 ± 12 aU). Prior PDTC application did not increase intestinal µflow during hemorrhagic shock or under physiological hemodynamic conditions. Blood transfusion led to a rapid recovery of µflow after 30 min in the ileum but not in the colon compared to baseline values. Whereas PDTC pretreatment accelerated the recovery of colonic µflow after blood transfusion, the effect on ileal µflow was contradictory. After 2 h of transfusion, µflow did not differ between the experimental groups. Likewise, hemorrhagic shock also led to a significant decrease of µvelo in the ileum (shock _ileum; 00:45_: 13 ± 3 aU) and in the colon (shock _colon; 00:45_: 14 ± 5 aU). Transfusion of the shed blood immediately reestablished baseline values of µvelo in both sections of the gastrointestinal tract. Prior PDTC application did not reveal a beneficial effect on intestinal µvelo (Fig. 8: Laser Doppler flowmetry).

Baseline MFI of the colon revealed intact microvascular perfusion in all experimental groups. Application of PDTC had no effect on colonic MFI under baseline conditions. Whereas MFI remained stable over time in normovolemic animals, acute hemorrhage led to a significant decrease of MFI from 3 ± 0 to 2.2 ± 0.1 compared to baseline values when measured one hour after the induction of fixed-pressure hemorrhage. This finding corresponds to a significant difference of MFI during hemorrhagic shock between normovolemic and hemorrhagic animals (control _colon; 01:15_: 3 ± 0; shock _colon; 01:15_: 2.2 ± 0.1). Prior PDTC treatment did not improve MFI during hemorrhagic shock (shock + PDTC _colon; 01:15_: 2.1 ± 0.1). Blood transfusion reestablished baseline MFI values in the shock groups. Final MFI values did not differ between experimental groups fully recovered after shed blood transfusion.

### Spatial microvascular perfusion characteristics

Baseline values of TVD (control _colon; 00:15_: 11 ± 1 mm · mm^− 2^), PVD (control _colon; 00:15_: 4.2 ± 0.6 mm · mm^− 2^) and PPV (control _colon; 00:15_: 39 ± 4) did not differ between experimental groups. There was a slight increase of TVD and PVD over time compared to baseline values. Neither prior PDTC application nor hemorrhage – shed blood transfusion affected TVD, PVD and PPV. At all timepoints of measurement, there was no difference between the experimental groups. HGI increased during acute hemorrhage (shock _colon; 01:15_: 0.1 ± 0.05) compared to the individual baseline (shock _colon; 01:15_: 0 ± 0) and the normovolemic control group (control _colon; 01:15_: 0.04 ± 0.04). Prior PDTC application further increased HGI during hemorrhagic shock (shock + PDTC _colon; 01:15_: 0.67 ± 0.1), but not in normovolemic animals (PDTC _colon; 01:15_: 0.09 ± 0.06).

### Intestinal tissue integrity

**Fig. 9:**
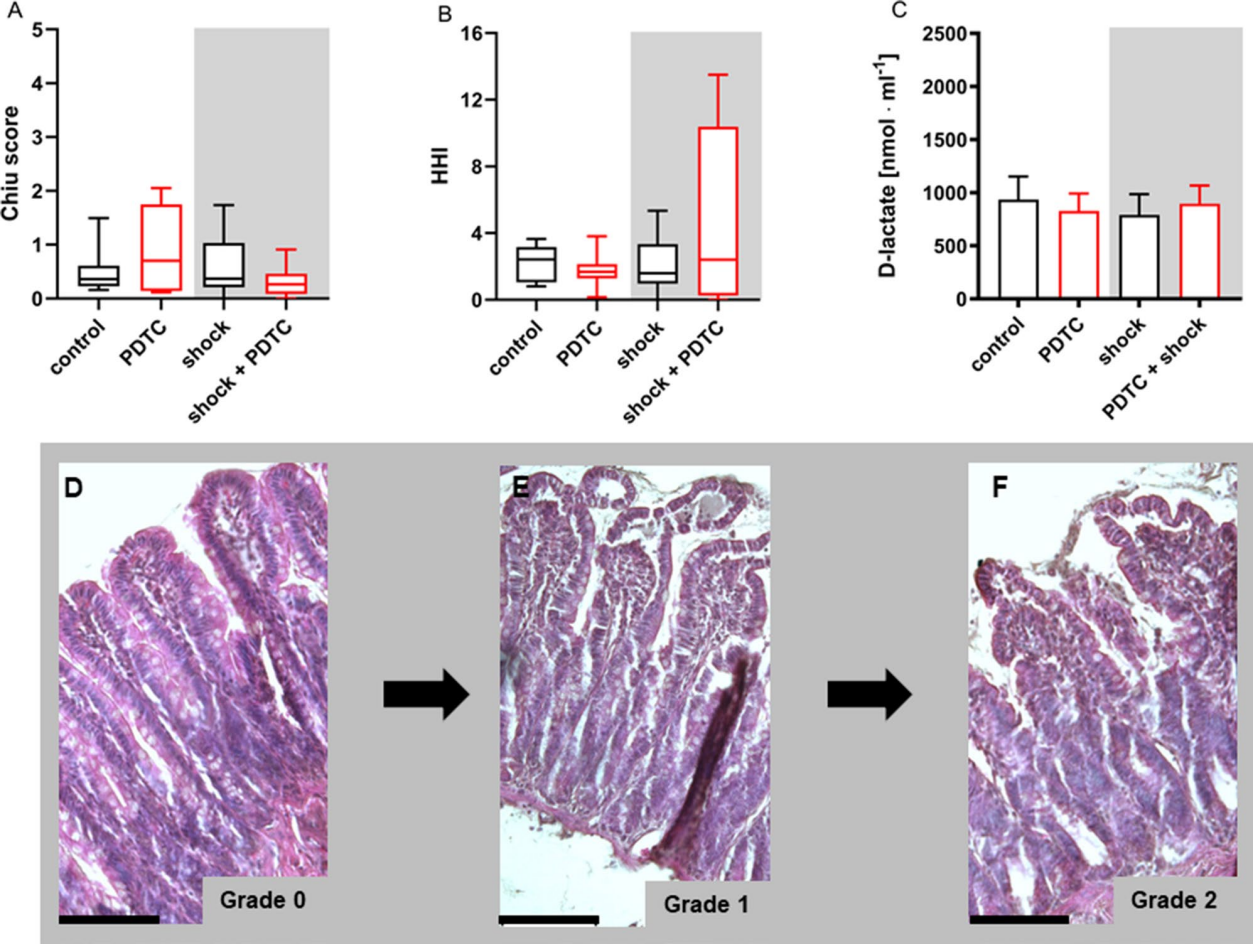
Intestinal tissue integrity. Intestinal tissue integrity was assessed by histological scoring (**A**; **B**) and plasmatic D-lactate concentration (**C**). Histological heterogeneity index was calculated as a measure of spatial tissue damage heterogeneity. Figure 9D-F are giving an impression of the most present histological injury patterns. Intestinal tissue structure was either maintained (**D**, scoring grade 0), slightly injured with apical (**E**, arrow, scoring grade 1) or extended (**E**, scoring grade 2) epithelial lifting from the lamina propia or progressively injured with progredient epithelial lifting and/or denuded stroma villi (**F**, scoring grade 3–4). All here shown stages of histological intestinal tissue damage were observed in all experimental groups. More severe injury patterns according to the Chiu scoring system were not observed. Black bars indicate distances of 100 μm

Neither structural integrity of the ileum, assessed by histological scoring, nor epithelial barrier function measured by plasmatic D-lactate concentration revealed a significant effect of shock induction or PDTC on intestinal tissue integrity, when measured in the end of the experiments (Fig. 9: Intestinal tissue integrity).

## Discussion

Acute hemorrhagic shock may induce severe tissue damage and multi-organ dysfunction by either microcirculatory impairment or insufficient mitochondrial respiration leading to regional tissue hypoxia. Pharmacological induction of HO-1 could be a promising target to protect tissues from distress and in turn might exert beneficial effects on mitochondrial respiration and microcirculation. This study was designed to expose the impact of PDTC pretreatment, that was reported to induce HO-1, as a pharmacological preconditioning strategy on intestinal microvascular oxygenation, mitochondrial function, microvascular perfusion markers and tissue integrity in a reversible model of hemorrhagic shock. The here obtained data support the following statements.

- The induction of acute hemorrhage leads to a depletion of intestinal oxygen reserve measured by µHbO_2_. Prior PDTC treatment improves colonic µHbO_2_ after 60 min of hemorrhagic shock and accelerates the recovery of intestinal µHbO_2_ in the early course of shed blood transfusion.
- The beneficial effects of PDTC pretreatment on colonic µHbO_2_ are independent of tissue heme oxygenase-1 induction.
- In this model of combined hemorrhage-reperfusion, mitochondrial function and oxidative stress remained unchanged, when assessed ex-vivo under standard conditions. This could be the result of maintained mitochondrial integrity in the here used shock model.
- Hemorrhagic shock is characterized by a reduction of intestinal microvascular perfusion. Prior PDTC treatment did not improve variables of microvascular blood flow.

Basically, tissue hypoxia is the leading distress of many shock forms and responsible for tissue injury and organ failure [49]. Therefore, we targeted µHbO_2_, a marker of postcapillary oxygen saturation and regional oxygen reserve, as the primary endpoint in this study. A decrease of gastrointestinal µHbO_2_ during hemorrhagic shock was reported to be associated with gastric [50] and intestinal [46] barrier dysfunction in previous studies, indicating early tissue injury during acute hemorrhage. Concordant to these findings, µHbO_2_ decreased after the induction of hemorrhagic shock in the recent study, indicating intestinal hypoxia and depletion of regional oxygen reserve during hemorrhagic shock. Whereas prior PDTC treatment had no effect on intestinal µHbO_2_ under physiological conditions, PDTC-pretreatment improved colonic µHbO_2_ during hemorrhagic shock. This effect was limited to the colonic tissue with no beneficial effect of PDTC treatment on ileal µHbO_2_. Comparing the trends of intestinal µHbO_2_ in normovolemic animals, we also observed a slight but continuous decrease of colonic µHbO_2_, whereas ileal µHbO_2_ remained unchanged over time. Contrary to studies suggesting that microvascular changes under restricted oxygen delivery are transferable between different regions of the gastrointestinal tract [51], our findings indicate that the microvascular response to anesthetics, hemorrhagic shock, and therapeutic interventions may vary between distinct intestinal segments. This is conclusive to previous studies of our group that have also shown differences of microvascular variables between two sections of the gastrointestinal tract when treated with topical drug application during acute hemorrhage [28, 36]. Divergences between the terminal ileum and the ascending colon are possibly a result of the section specific vascular organization of the supplying blood vessels [52]. Whereas colonic vessels are reported to be functional end arteries, the vascular arcades of the small intestine are formatting collaterals between adjacent vascular units. This might enable microvascular cross-flow in the small intestine under conditions of microvascular blood flow disturbance leading to inconclusive results between ileal and colonic measurements. The two intestinal regions not only differ in their vascular blood supply but also in their functional roles, which results in varying physiological demands. These distinctions may lead to differential activation of metabolic pathways, distinct stress responses, and varying oxygen consumption. One indication that both investigated sections of the gastrointestinal tract differ not only in their vascular blood supply but also in their cellular function is the distinct expression of HO-1 after PDTC pretreatment.

In this study relative protein induction of HO-1 increased in the ileum after PDTC treatment underlining biological activity of the here used PDTC concentration. The concentration and the time point of PDTC application were adapted from experimental studies that used PDTC to successfully induce HO-1 in intestinal tissue [34, 53, 54]. Surprisingly, colonic µHbO_2_ of animals with PDTC application improved during hemorrhagic shock without a relative increase of HO-1 tissue protein expression. On the one hand this could be related to HO-1 independent effects of PDTC. PDTC was reported to exert general immunomodulation by inhibition of NF-ĸB and subsequent depression of proinflammatory genes in a murine model of septic shock [55]. NF-ĸB inhibition by PDTC appears to depend on the regulator of calcineurin activity-1 (RCAN-1) [56] and modulates the immunological effect of IL-6 [57], TNF-α [58] and other proinflammatory mediators. In addition, PDTC leads to an upregulated expression of occludin and zonulin-1 in mice with DSS colitis [59]. Both, reducing local inflammation and upregulating cell adherence proteins could enhance the reliance of intestinal tissue against hemorrhagic shock and hypoxic tissue damage without an activation of heme oxygenase-1. Whether these effects are also linked to a reduction in cellular energy demand has not been investigated yet, but could explain the beneficial effects of PDTC on µHbO_2_. It should be emphasized that our study was not conceptualized to investigate HO-1 dependent and HO-1 independent effects of PDTC on µHbO_2_. In addition, the presented link is not directly supported by the data presented in this study. On the other hand, there was a clear optical native signal of HO-1 in colonic tissue of animals, that did not receive PDTC. A continuous expression of HO-1 in the colon could serve as an adaptive mechanism to counteract adverse stimuli derived from colonic enterobacteria but this hypothesis remains speculative [60]. However, a continuous colonic HO-1 expression under physiological conditions could reduce the relative increase of HO-1 protein expression after PDTC pretreatment. Taking these aspects together, changes in HO-1 protein expression by PDTC and its effect on µHbO_2_ during hemorrhagic shock appear to depend on the investigated tissue section of the gastrointestinal tract.

Illuminating the impact of PDTC application on intestinal µHbO_2_ in the context of resuscitation, PDTC appears to accelerate the recovery of local oxygen reserve in both sections of the gastrointestinal tract after shed blood transfusion. Oxidative stress is a major component of reperfusion injury after ischemic events [61] and HO-1 was reported to play a pivotal role in the control of oxidative balance [62]. Therefore, HO-1 induction by PDTC application could reduce oxidative stress during resuscitation leading to improved regional oxygen metabolism. It has to be mentioned, that beneficial effects of PDTC pretreatment on intestinal µHbO_2_ were limited to the late course of hemorrhagic shock and the initial period of resuscitation. Two hours after shed blood transfusion intergroup analysis did not reveal a significant effect of PDTC pretreatment on intestinal µHbO_2_ and regional tissue MDA concentration. Despite favorable effects in the early period of resuscitation, it therefore has to be clarified in further studies, if prior PDTC treatment also leads to a long-term benefit after hemorrhagic shock. However, the results indicate that PDTC administration during the acute shock phase and the initial resuscitation period exerts beneficial effects, highlighting molecular pathways and mechanisms that may inform future pharmacological strategies to support existing therapeutic approaches. Since the effect of shed blood transfusion on systemic hemodynamics, microvascular perfusion, and tissue oxygenation were more pronounced than those of prior PDTC administration we also anticipate, that PDTC cannot replace blood transfusion in the context of hemorrhagic shock. However, it may beneficially modulate the immediate effects of red blood cell transfusion.

Since monitoring intestinal tissue damage still remains challenging in the clinical and the experimental setting, hemorrhagic shock with subsequent transfusion had no effect on histological scoring of the intestine and plasmatic D-lactate concentration in the current study. Histological scoring like described by Chiu et al. is mainly used in models of complete vascular ischemia with reperfusion to visualize structural damage to the intestinal tissue [63, 64]. Hemorrhagic shock with subsequent transfusion fundamentally differs from ischemia reperfusion respective the occurrence of residual microvascular blood flow. It is therefore unclear whether the results of histological evaluation can be transferred from animals with ischemia reperfusion to models of hemorrhagic shock and transfusion. In addition, most of the studies using histological scoring to evaluate intestinal damage harvested tissue samples 24 h after the onset of shock [65, 66]. Probably, histologically visible damage to the intestine develops after several hours and cannot be observed within the first hours after shock. In accordance to the concept of delayed histological assessment of intestinal tissue injury, plasmatic D-lactate concentration as a marker of intestinal barrier dysfunction mirrored intestinal tissue injury more accurately after one hour of acute isolated hemorrhagic shock than histological staining in one of our previous studies [46]. We concluded, that plasmatic D-lactate could serve as an early plasmatic marker of intestinal barrier dysfunction. Plasmatic D-lactate concentration did not increase in animals with shock in the recent study. This could be attributed to a dilution of plasmatic D-lactate by shed blood transfusion. Since D-lactate is a product of enterobacterial metabolism, there was no enrichment of the shed blood with D-lactate during the shock period ex-vivo. These results are in accordance with earlier studies on plasmatic D-lactate concentration, that reported plasmatic D-lactate concentration to be accurate to indicate shock depth in animals undergoing hemorrhagic-traumatic shock. In this study, there was also a reduction of plasmatic D-lactate concentration after hemodynamic resuscitation using shed blood transfusion [67]. Thus, the exact role of D-Lactate as early marker of intestinal tissue damage in the used model of hemorrhage with transfusion needs further evaluation. Taken together, these results support the use of µHbO_2_ as an early dynamic marker of microvascular oxygenation to identify gastrointestinal tissue at risk for further damage in models of hemorrhagic shock. PDTC pretreatment improves µHbO_2_ during late hemorrhagic shock and early resuscitation. These effects of PDTC are possibly independent of local HO-1 induction. It has to be clarified in further studies, if these findings are leading to long-term tissue protection of intestinal tissue. The measurement of plasmatic D-lactate concentration and the histological evaluation of intestinal tissue integrity probably cannot be used in models of hemorrhagic shock with subsequent shed blood transfusion to monitor intestinal tissue injury in the acute or subacute course of critical illness.

µHbO_2_ as a measure of regional oxygen reserve may be subject to confounders such as shunting on a microvascular or mitochondrial level. Therefore, we further investigated the effect of prior PDTC treatment on variables of mitochondrial function and intestinal microvascular perfusion. Previously, we reported that isolated hemorrhagic shock does not influence mitochondrial respiration rates, mitochondrial efficiency and coupling between the respiratory chain and the oxidative phosphorylation when measured ex-vivo by respirometry [46]. Reperfusion injury could contribute significantly to mitochondrial dysfunction like described in models of myocardial [68, 69] and cerebral [70] ischemia-reperfusion and would qualify mitochondrial respiration as a target for pharmacological intervention by PDTC. However, neither the induction of hemorrhagic shock with subsequent reperfusion, nor prior PDTC treatment revealed a significant effect on mitochondrial function in this study. Obviously, mitochondrial integrity is generally maintained in this model of hemorrhage-reperfusion giving the opportunity to reestablish adequate mitochondrial respiration under physiological conditions ex-vivo. Hemorrhagic shock significantly reduced microvascular perfusion measured by µflow and µvelo in both sections of the intestine. Further colonic MFI as a measure of qualitative microvascular blood flow characteristic decreased after the induction of acute hemorrhage. These findings were in accordance with our previous results on microvascular variables during hemorrhagic shock [28, 36, 37, 50]. Transfusion of the shed blood reestablished baseline values of all perfusion markers with no differences between the experimental groups after two hours of transfusion. Since mitochondrial function was maintained after isolated hemorrhagic shock [46] and after hemorrhagic shock with subsequent transfusion as can be deduced from the current study, intestinal hypoxia and depletion of the regional oxygen reserve appears to be primary a result of reduced microvascular perfusion without mitochondrial failure in the acute phase of hemorrhagic shock. Therefore, resuscitation of the microvascular perfusion should be one major goal of early resuscitation strategies for patients suffering from hemorrhagic shock. However, in colon and ileum, PDTC pretreatment failed to improve µflow and µflow during hemorrhagic shock. In contrast, PDTC was reported to improve intestinal microvascular perfusion in models of ischemia reperfusion [34, 53, 54]. Thus, the effect of PDTC on microvascular perfusion might depend on the injury model used to compromise intestinal microvascular perfusion. The MFI appears to be a promising marker of deranged microvascular perfusion in clinical trials, since lower values of the MFI were associated with increased morbidity and mortality in patients with traumatic hemorrhage [71, 72] and in a mixed ICU population [73, 74]. Further, enhanced sublingual MFI during hemorrhagic shock was associated with improved tissue oxygenation measured by µHbO_2_ in one of our previous studies [36]. In this study, not only µflow and µvelo but also MFI-values in hemorrhagic animals were not altered by prior PDTC application. In accordance, TVD, PVD and PPV did not differ between experimental groups and were neither influenced by the induction of hemorrhagic shock nor by PDTC pretreatment.

In this study, we promoted systemic PDTC application as a promising strategy for intestinal tissue protection after severe trauma and hemorrhagic shock. If the here described effects of PDTC on regional tissue oxygenation are transferable to other organs at persistent risk for tissue injury and organ dysfunction in the context of hemorrhagic shock and trauma has to be clarified. Furthermore, it has to be mentioned that PDTC was applied 24 h before the onset of hemorrhagic shock. One should consider that severe trauma with accompanying hemorrhage is frequently an unpredictable event. Therefore, the concept of pharmacological preconditioning by PDTC is currently limited to the context of perioperative care where acute bleeding can be anticipated in patients with planned major surgery. The more it is of outstanding interest to elucidate the subordinated mediators and pathomechanisms of PDTC related tissue protection to identify possible targets of pharmacological conditioning in the acute phase of trauma and hemorrhagic shock.

## Conclusion

Taken together, the application of PDTC leads to an improved intestinal oxygen reserve especially during shed blood transfusion but also during late hemorrhagic shock of the colon. Maintaining adequate tissue oxygenation is one of the most important goals to prevent multi organ dysfunction in critically ill patients. Therefore, these findings could be of crucial relevance to identify new therapeutic targets to support global homeostasis in patients suffering from hemorrhagic shock. The mechanisms leading to improved tissue oxygenation after PDTC pretreatment are unclear, as in this study there is neither an optimization of mitochondrial function nor an improvement of reduced intestinal perfusion during hemorrhagic shock. Further, it is unclear if the beneficial effects of PDTC exclusively depend on increased HO-1 expression, since colonic HO-1 expression remained unchanged after PDTC stimulation. HO-1 dependent and HO-1 independent effects of PDTC should be investigated in further studies to identify possible targets for gastrointestinal tissue protection during hemorrhagic shock. Conclusively, PDTC treatment appears to be a promising concept of pharmacological preconditioning in the context of hemorrhagic shock with subsequent shed blood transfusion.

## Data Availability

The dataset used and analysed during the study are available from the corresponding author on reasonable request.

## Abbreviations

HGI: Heterogeneity index
HO-1: Heme oxygenase-1
IDF: Incident dark-field
MAP: Mean arterial blood pressure
MDA: Malondialdehyde
MFI: Microvascular flow index
µflow: Microvascular blood flow
µHbO_2_: Microvascular oxygenation
µvelo: Microvascular blood flow velocity
PDTC: Pyrrolidine dithiocarbamate
PPV: Proportion of perfused vessels
PVD: Perfused vessel density
RCI: Respiratory control index
TVD: Total vessel density
